# Epigenetic control of phospholipase A_2_ receptor expression in mammary cancer cells

**DOI:** 10.1186/s12885-015-1937-y

**Published:** 2015-12-16

**Authors:** Mario Menschikowski, Albert Hagelgans, Brit Nacke, Carsten Jandeck, Olga Sukocheva, Gabriele Siegert

**Affiliations:** 1 Institute of Clinical Chemistry and Laboratory Medicine, Medical Faculty “Carl Gustav Carus”, Technical University of Dresden, Fetscherstr. 74, 01307 Dresden, Germany; 2 School of Health Sciences, Flinders University of South Australia, Bedford Park, SA 5042 Australia

## Abstract

**Background:**

It has recently been proposed that the M-type phospholipase A_2_ receptor (PLA2R1) acts as a tumour suppressor in certain malignancies including mammary cancer. Considering that DNA methylation is an important regulator of gene transcription during carcinogenesis, in the current study we analyzed the PLA2R1 expression, *PLA2R1* promoter methylation, and selected micro RNA (miRNA) levels in normal human mammary epithelial cells (HMEC) and cancer cell lines.

**Methods:**

Levels of PLA2R1 and DNA methyltransferases (DNMT) specific mRNA were determined using real-time RT-PCR. Methylation specific-high resolution melting (MS-HRM) analysis was utilized to quantify the methylation degree of selected CpG sites localized in the promoter region of the *PLA2R1* gene. Expression of miRNA was tested using miScript Primer Assay system.

**Results:**

Nearly complete methylation of the analyzed *PLA2R1* promoter region along with *PLA2R1* gene silencing was identified in MDA-MB-453 mammary cancer cells. In MCF-7 and BT-474 mammary cancer cell lines, a higher DNA methylation degree and reduced PLA2R1 expression were found in comparison with those in normal HMEC. Synergistic effects of demethylating agent (5-aza-2′-deoxycytidine) and histone deacetylase inhibitor (trichostatin A) on PLA2R1 transcription in MDA-MB-453 cells confirmed the importance of DNA methylation and histone modification in the regulation of the *PLA2R1* gene expression in mammary cells. Furthermore, significant positive correlation between the expression of DNMT1 and *PLA2R1* gene methylation and negative correlation between the cellular levels of *hsa-mir-141, −181b, and -181d-1* and the expression of PLA2R1 were identified in the analyzed cells. Analysis of combined z-score of *miR-23b, −154 and -302d* demonstrated a strong and significant positive correlation with PLA2R1 expression.

**Conclusions:**

Our data indicate that (i) PLA2R1 expression in breast cancer cells is controlled by DNA methylation and histone modifications, (ii) hypermethylation of the *PLA2R1* promoter region is associated with up-regulation of DNMT1, and (iii) *hsa-miR-23b, −154, and −302d*, as well as *hsa-miR-141, −181b, and −181d-*1 are potential candidates for post-transcriptional regulation of PLA2R1 expression in mammary cancer cells.

## Background

M-type phospholipase A_2_ receptor (PLA2R1) is a 180 kDa transmembrane glycoprotein that belongs to the C-type lectin superfamily and the mannose receptor family. PLA2R1 consists of cystein-reach domain, fibronectin type II domain and eight carbohydrate recognition domains [1, 2]. The receptor binds secreted phospholipases A_2_ (sPLA_2_) with distinct affinities [3, 4]. As result of sPLA_2_ binding to PLA2R1, the amount of sPLA_2_ is lowered in extracellular milieu and its cellular signaling cascades linked to apoptosis and senescence are switched on [4]. A soluble form of the receptor is constitutively present in circulation as an endogenous inhibitor for mammalian sPLA_2_s [3].

Limited number of studies addressed the pathophysiological role of PLA2R1. It has been shown that PLA2R1 is the major podocyte autoantigen associated with development of idiopathic membranous nephropathy [5, 6]. Anti-PLA2R1 autoantibodies bind to conformational epitopes on the receptor, form immune complexes which stimulate the release of cytokines and metalloproteinases; that, in turn, result in proteinuria [5–7]. Recent studies uncovered a novel tumour suppressive function of PLA2R1. The receptor exerted anti-tumour responses including cellular senescence, apoptosis and inhibition of cell transformation [8–11]. For instance, the ability of human mammary epithelial cells (HMEC) to overcome oncogenic stress–induced senescence was improved after downregulation of PLA2R1 levels *in vitro* [10]. Furthermore, in mammary cancer cell lines MDA-MB-231 and Cama-1 the constitutive expression of PLA2R1 was found to block the colony growth in soft agar, supporting a tumour suppressive role of PLA2R1 [10]. Contrary, knockdown of PLA2R1 increased the transformed phenotype of MDA-MB-436 breast cancer cells as measured by the increased size of soft agar colonies. In addition, *PLA2R1*-deficient mice displayed increased sensitivity to RAS-induced tumourigenesis by facilitating oncogenic stress-induced senescence escape in vivo, highlighting the role of the receptor as tumour suppressor [10].

PLA2R1 expression was found decreased in leukaemia, mammary, renal and thyroid cancers [9, 12–15]. While the receptor is down-regulated in these cancers, significant up-regulation of PLA2R1 was described in the prostate cancer cell lines PC-3 and DU-145 in comparison to normal prostate cells [16]. High expression of PLA2R1 was also identified in ovarian carcinoma effusions, human leukemic blasts and dermatofibrosarcoma [17–19]. However, detailed functions and mechanisms of PLA2R1-mediated signalling in normal and different cancer cells remain to be elucidated.

It is well-known that epigenetic mechanisms play a crucial role in cell reprogramming during carcinogenesis. DNA methylation, histone modification, and posttranscriptional gene regulations by non-coding RNAs (microRNAs, long non-coding RNAs, and small nuclear RNAs) were also detected at earlier stages of neoplastic transformation essential for cancer initiation and progression [20]. We have recently detected *PLA2R1* promoter hypermethylation in leukemic cell lines and leukocytes of patients with leukemia [12]. More hypermethylations of CpG sites in the *PLA2R1* promoter region were recently found in PLA2R1-negative kidney cell lines compared to PLA2R1-positive cells [14]. To decrease the tumour suppressive effect, cancer cells may exploit hypermethylation of the *PLA2R1* promoter as gene silencing mechanism [12].

The purpose of this study was to examine expression of PLA2R1, degree of *PLA2R1* promoter methylation, and expression of methylation regulating enzymes DNA-methyltransferases (DNMT) in normal and mammary cancers cell lines. Levels of distinct miRNAs that may target PLA2R1 mRNA were also assessed. Correlations among expression of *PLA2R1*, degree of *PLA2R1* gene methylation and related miRNAs were tested.

## Methods

### Cell culture and treatments

Human mammary epithelial cells (HMEC) were from Lonza (Köln, Germany) and the human UACC-812 and MCF-7 mammary cancer cell lines were from the American Type Culture Collection (Rockville, MD, USA). Additional human mammary cancer cell lines, Cal-51, BT-474 and MDA-MB-453, were obtained from the German Collection of Microorganisms and Cell Cultures (Berlin, Germany). HMEC were cultured in MEGM culture medium and MCF-7 cells in RPMI 1640 culture medium supplemented with 10 % FCS at 37 °C in a humidified atmosphere of 5 % CO_2_. Cal-51, BT-474 and MDA-MB-453 cell lines were cultured in L-15 (Leibovitz) medium (Sigma-Aldrich) supplemented with 20 % FCS and incubated at 37 °C under conditions of free gas exchange with atmospheric air. All cells were incubated in the presence of 1 % penicillin/streptomycin (Invitrogen) and 0.36 % gentamycin (Invitrogen). Clinicopathological and biological characteristics of the analyzed cell lines were described in details elsewhere [21–23].

To estimate the role of epigenetic mechanisms in PLA2R1 expression, 5-aza-2′-deoxycytidine and trichostatin A (TSA, Sigma-Aldrich; Deisenhofen, Germany) were used as described previously [24]. MDA-MB-453 cells were seeded at a density of 5 × 10^5^ cells per well into 24-well tissue culture plates 24 h before 5-aza-dC and TSA treatments. Cells were treated with 1 μM 5-aza-dC for 72 h and 0.3 μM TSA for 24 h alone and in combination. During combined treatment, cells were exposed first to 1 μM 5-aza-dC for 48 h and then to 0.3 μM TSA for the following 24 h together with 5-aza-dC. After incubation, cells were harvested and DNA and RNA were isolated for MS-HRM and real-time RT-PCR analyses.

### Extraction of genomic DNA and RNA

Genomic DNA and RNA were isolated from normal HMEC and mammary cancer cell lines using the Blood & Cell Culture DNA Mini Kit from Qiagen GmbH (Hilden, Germany) and TRI Reagent from Sigma-Aldrich according to the manufacturer’s instructions.

### Analysis of miRNA expression

Micro RNAs (miRNA) were isolated from normal and cancer cells using the miRNeasy Mini and RNeasy MinElute Cleanup Kits (Qiagen GmbH) according to manufacturer’s instructions. The expression of miRNAs was analyzed using the miScript Primer Assay system (Qiagen GmbH) with the Rotor-Gene Q (Qiagen GmbH). Data were analyzed using the comparative quantification method wherein relative levels of miRNA were normalized to non-coding small nuclear RNA U6 (U6 snRNA) level. The following miScript Primer Assays were used: MS00031633 (Hs_miR-23a_2), MS00031647 (Hs_miR-23b_2), MS00022897 (Hs_miR-23c_1), MS00003507 (Hs_miR-141_1), MS00003570 (Hs_miR-149_1), MS00003598 (Hs_miR-154_1), MS00006699 (Hs_miR-181b_1), MS00045969 (Hs_miR-181d-3p_1), MS00031500 (Hs_miR-181d_2), MS00003920 (Hs_miR-302d_1), MS00009835 (Hs_miR-501-5p_1) and MS00033740 for U6 snRNA (Hs_RNU6-2_11).

### Quantitative RT-PCR analyses

Isolated RNA was converted to cDNA using the GeneAmp RNA-PCR Kit (PerkinElmer LAS GmbH, Jügesheim, Germany). For quantitative RT-PCR, portions of the reverse transcribed reaction products were amplified for identification of PLA2R1 expression comparing to GAPDH levels used as reference gene. Real-time RT-PCR was performed using Rotor-Gene Q and Rotor Gene SYBR Green PCR kit (Qiagen GmbH) according to manufacturer’s instructions. The primer pairs used for the analyses of GAPDH and PLA2R1 expression were: GAPDH, forward 5′-CGG AGT CAA CGG ATT TGG TCG TAT TG-3′ and reverse 5′-GCA GGA GGC ATT GCT GAT GAT CTT G-3′ giving PCR products with a length of 439 bp [25]; PLA2R1, forward 5′-CAG AAG AAA GGC AGT TCT GGA TTG-3′ and reverse 5′-AAA GCC ACA TCT CTG GCT CTG ATT-3′ for PLA2R1, giving PCR products with a length of 325 bp. DNA methyltransferases primer sequences were: DNMT1, forward 5′-GTG GGG GAC TGT GTC TCT GT-3′ and reverse 5′-TGA AAG CTG CAT GTC CTC AC-3′ giving PCR product with a length of 204 bp; DNMT3A, forward 5′-CCA GTT AGC AGC AGG GAG AC-3′ and reverse 5′-CAA GAG GTA ACA GCG GCT TC-3′ giving PCR product with a length of 119 bp and DNMT3B, forward 5′-CAG GGA AAA CTG CAA AGC TC-3′ and reverse 5′-ATT TGT TAC GTC GTG GCT CC-3′ giving PCR product with a length of 296 bp. Primers were applied in a final concentration of 0.8 μM. The conditions for amplification were as follows: 40 courses at 95 °C for 5 s and 58 °C for 10 s. At the beginning of real-time RT-PCR analyses, the size and purity of the amplification products were confirmed using agarose gel electrophoresis.

### Methylation-specific high resolution melting (MS-HRM) analyses

MS-HRM analyses were conducted to quantify the degree of methylation in the distinct region from −437 bp to −270 bp of exon 1 of the *PLA2R1* gene (ENSG00000153246, transcript: PLA2R1-001 ENST00000283243). The analyses were performed using Rotor-Gene Q and the EpiTect MS-HRM PCR kit (Qiagen GmbH) according to manufacturer’s instructions. The applied primer pairs for *PLA2R1,* standards and additional details of the MS-HRM analyses were described previously [12, 26]. Briefly, bisulfite modified unmethylated and methylated standard DNA (Qiagen GmbH) were mixed giving samples with 0, 10, 25, 50, 75, and 100 % methylation degrees for calibration. A standard curve with known methylation degrees was included in each run. The applied primer pairs for *PLA2R1* were 5′-GGG GTA AGG AAG GTG GAG AT-3′ and 5′-ACA AAC CAC CTA AAT TCT AAT AAA CAC-3′ giving PCR products with a length of 168 bp. The primers were applied at a final concentration of 0.8 μM. The conditions of amplification were as follows: 40 courses at 95 °C for 10 s, 58 °C for 30 s and 72 °C for 15 s. Immediately after PCR, the products were analyzed by high resolution melting analysis with fluorescence measured during the linear temperature transition from 50 to 95 °C at 0.01 °C/s.

### *In silico* analyses

MethPrimer software [27] was used to establish primers for MS-HRM and assess the presence of 5′-CpG islands in the promoter region of the *PLA2R1* gene. Prediction of putative binding sites for transcription factors in the *PLA2R1* promoter region was performed using Promo software V.3 [28]. Search of candidate miRNAs which might target the *PLA2R1* gene expression was performed using following prediction programs: miRDB (http://mirdb.org/cgi-bin/search.cgi), microRNA.org-Targets and Expression (http://www.microrna.org). Data were also assessed using MirWalk program, which includes information about miRNA target interactions produced by established miRNA prediction programs on 3′ UTRs of all known human, mouse and rat genes [RNA22, miRanda, miRDB, TargetScan, RNAhybrid, PITA, PICTAR, and Diana-microT (http://www.umm.uni-heidelberg.de/apps/zmf/mirwalk/predictedmirnagene.html)].

### Data analysis

The differences between the studied groups were analyzed with Kruskal-Wallis one way test of variance on ranks. The correlations between variable pairs were studied using Pearson product moment correlation test. All statistical analyses were performed using the statistics module integrated in the SigmaPlot 11.2 software (Systat Software GmbH, Erkrath, Germany). Differences were considered significant at *p* < 0.05.

## Results

**Fig. 1:**
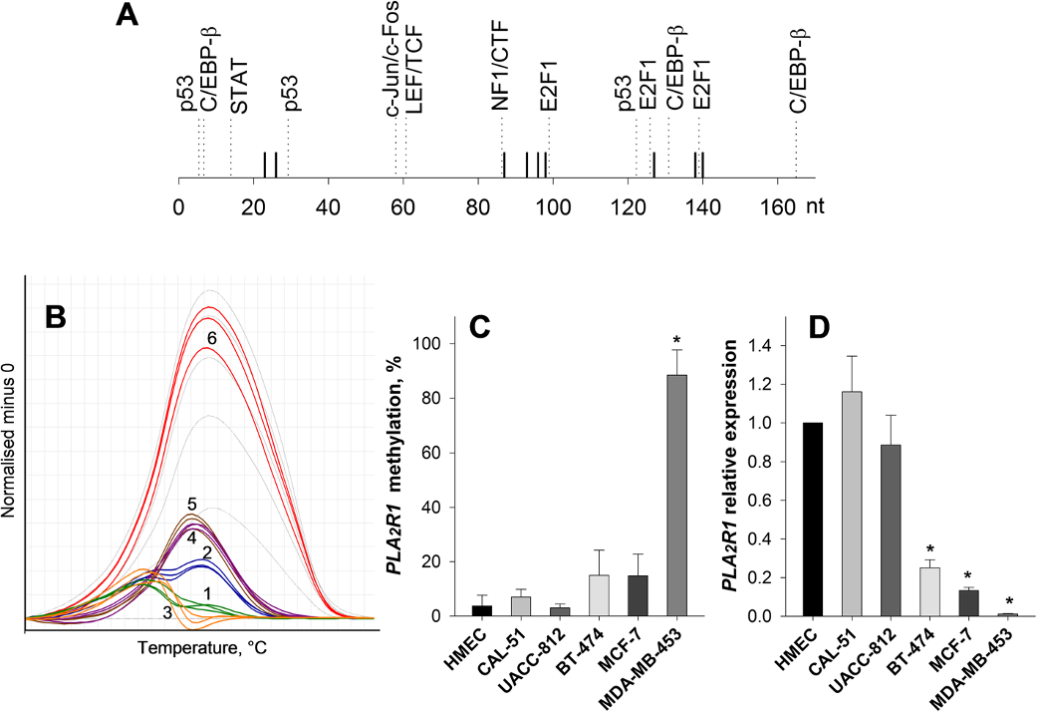
PLA2R1 expression and *PLA2R1* promoter methylation in HMEC and mammary cancer cell lines. **a** Part of the proximal promoter region of human *PLA2R1* gene (ENSG00000153246, transcript is shown. PLA2R1-001 ENST00000283243) expands from −437 to −270 bp relative to exon-1. The sequence in which the methylation degree was quantified using MS-HRM analysis contains nine CpG sites. Positions of potential transcription factor binding sites in the proximal part of the *PLA2R1* gene are indicated. **b** Difference plots normalized to the 0 %-methylated standard DNA sample and a standard curve with 0, 10, 25, 50, 75, and 100 % methylation ratios in black dotted lines (from the bottom up) are shown. Cell lines (1–6): HMEC, CAL-51, UACC-812, BT-474, MCF-7 and MDA-MB-453, respectively. **c** Bar graphs show the mean *PLA2R1* methylation degree *±* SD (%) calculated from two MS-HRM analyses in triplicates*.* **d** Relative expression levels of PLA2R1 and GAPDH as reference gene were determined in HMEC and mammary cancer cells using real-time RT-PCR. The estimated values of comparative quantification were normalized to levels of expression in HMEC that was set at 1.0. Results are shown as means *±* SD*.* Analyses were performed in duplicates and experiments were repeated three times. * - *p* < 0.05 relative to HMEC values

Using MS-HRM and *in silico* analyses we identified potential transcription factor binding sites in the PLA2R1 promoter region. We suggested that the region from −270 bp to −437 bp might contain CpG sites for E2F-1 and NFI/CTF or those located near CpG sites for C/EBP-β, p53, c-Jun/c-Fos, and LEF/CTF (Fig. 1a). MS-HRM analyses demonstrated differential *PLA2R1* promoter methylations in HMEC and breast cancer cell lines (Fig. 1b and c). In normal cells and CAL-51 and UACC-812 cancer cell lines the *PLA2R1* methylation degree was negligible (<10 %), whereas in MDA-MB-453 cells it reached a value of about 80 %. Simultaneously, MDA-MB-453 cells demonstrated strongly downregulated levels of the receptor mRNA representing only ~1 % of those in HMEC (Fig. 1d). In BT-474 and MCF-7 cells levels of PLA2R1-specific mRNA were also markedly reduced, whereas in CAL-51 and UACC-812 cells, levels of PLA2R1-specific mRNA were similar to those in normal HMEC (Fig. 1d). The Pearson product moment correlation coefficient between PLA2R1 expression and *PLA2R1* promoter methylation reached −0.664 but the relationship was not statistically significant (Table 1).

**Table 1:** Pearson product moment correlation coefficients (*r*) and significance (*p*) between PLA2R1 expression and variables such *PLA2R1* promoter methylation and means of z-score transformed data of candidate miRNAs expression in HMEC and mammary cancer cell lines (CAL-51, UACC-812, BT-474, MCF-7, and MDA-MB-453) are shown. Correlations were analysed using statistical package of SigmaPlot 11.2 software

Variables	Correlation with PLA2R1 expression	
	*r*	*p*
*PLA2R1* promoter methylation	−0.664	0.150
Mean z-score of all miRNAs	−0.077	0.885
Mean z-score of *hsa-mir-141, −149, −181b, −181d-1, −501*	−0.581	0.227
Mean z-score of *hsa-mir-141, −181b, −181d-1*	−0.726	0.102
Mean z-score of *hsa-mir-23b, −154, −302d*	0.923	0.009

**Fig. 2:**
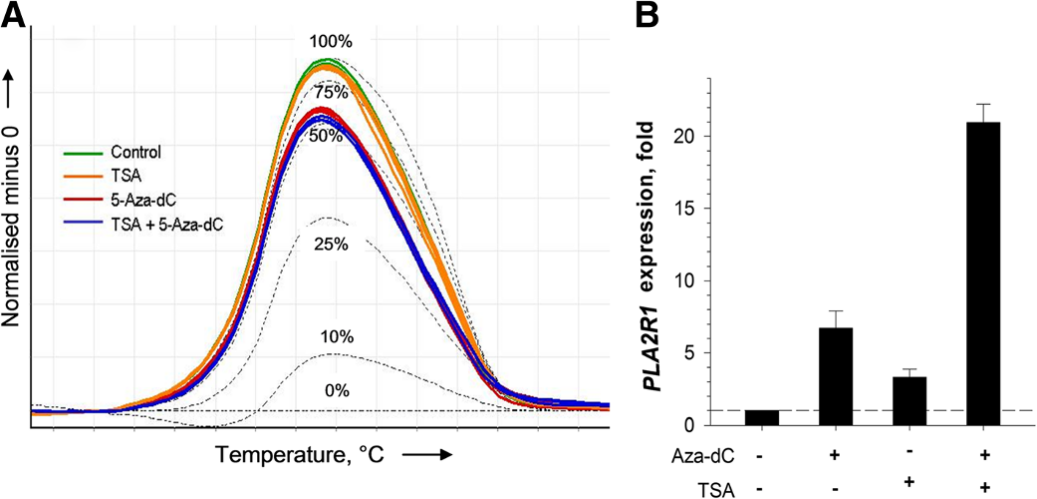
Effects of 5-aza-2′-deoxycytidine (5-aza-dC) and trichostatin A (TSA) treatments on *PLA2R1* promoter methylation (**a**) and PLA2R1 expression (**b**) in MDA-MB-453 cancer cells. **a** MS-HRM analysis of *PLA2R1* methylations in control cells without treatment (one, *green*) and after exposure of MDA-MB-453 cells to 1 μM 5-aza-dC for 72 h (two, *red*), 0.3 μM TSA for 24 h (three, *orange*), and in cells treated with both agents (four, *blue*) as described in Materials and Methods section. Difference plots were normalized to standard 0 %-methylated DNA sample data. The black dotted lines represent standard xcurves of 0, 10, 25, 50, 75 and 100 % methylation degrees. Analyses were performed in duplicates and results are representative of two independent experiments. **b** Relative expression of PLA2R1-specific mRNA was determined using real-time RT-PCR and GAPDH-mRNA levels were used as reference in MDA-MB-453 cells. The experimental conditions were the same as described in (**a**). Values of comparative quantification were normalized to expression levels of untreated controls that were set at 1.0. Results are shown as means *±* SD*.* Analyses were performed in triplicates and results are representative of two independent experiments

To examine the role of DNA methylation and histone modifications in silencing the *PLA2R1* gene expression MDA-MB-453 cells were exposed to demethylating agent, 5-aza-dC and histone deacetylase inhibitor, TSA. MS-HRM analyses revealed a nearly 50 % reduction of *PLA2R1* gene methylation degree with 1.0 μM 5-aza-dC for 72 h, whereas 0.3 μM TSA for 24 h as expected had a negligible effect on *PLA2R1* promoter methylation in comparison to untreated control cells (Fig. 2a). Treatment of cells with 5-aza-dC or TSA alone was accompanied by 6.7- and 3.3-fold increased PLA2R1 transcript levels, respectively (Fig. 2b). The combined application of both agents resulted in a synergistic 20.9-fold re-expression of PLA2R1 relative to untreated control cells, suggesting that, in addition to DNA methylation, histone deacetylation plays an important role in the regulation of PLA2R1 expression in MDA-MB-453 cells (Fig. 2b).

**Fig. 3:**
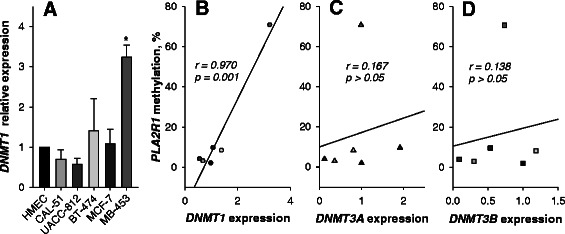
Expression of DNMT1 (**a**) and correlation of DNMT transcript levels with *PLA2R1* gene methylation degrees (**b**–**d**) in HMEC and mammary cancer cell lines. **a** Bar graphs show the relative levels of DNMT1-specific mRNA determined using real-time RT-PCR and GAPDH mRNA levels were used as reference gene. The comparative values were normalized to levels of DNMT1 expression in HMEC that was set at 1.0. Results are shown as means *±* SD*.* Analyses were performed in duplicates and results are representative of three independent experiments. * - *p* < 0.05 relative to HMEC value. **b**–**d** Data represent scatter-regression plots of correlation between *PLA2R1* promoter methylation and DNMT1 (**b**), DNMT3A (**c**) and DNMT3B (**d**) expressions in HMEC and mammary cancer cell lines. Values of Pearson product moment correlation coefficient (*r*) between DNMT expression and *PLA2R1* promoter methylation are shown

Next, we analyzed expression levels of DNMT1, the primary enzyme responsible for copying methylation patterns after DNA replication [29]. The analysis revealed significantly increased levels of DNMT1 transcripts in MDA-MB-453 cells with the highest *PLA2R1* gene methylation followed by the BT-474 and MCF-7 cell lines (Fig. 3a). The *PLA2R1* methylation degree in mammary cells strongly correlated with DNMT1 expression level (*r* = 0.970; *p* = 0.001, Fig. 3b). In contrast, levels of DNMT3A- and DNMT3B-specific mRNA were not significantly associated with *PLA2R1* gene methylation (Fig. 3c and d).

**Fig. 4:**
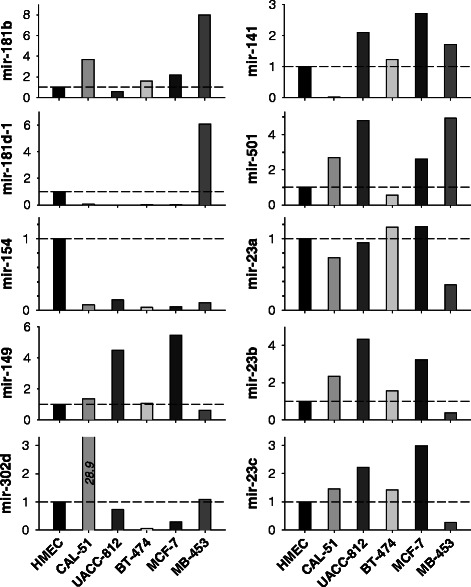
Values of miRNAs expression in mammary cancer cell lines normalized to mean values of that in normal HMEC. Bar graphs demonstrate miRNA levels in cancer cells relative to HMEC that was set at 1.0. Details of miRNA quantification are described in the Materials and Methods section. Means ± SD values of miRNAs expression are shown in Table 2

Using miRNA prediction software, we identified a number of PLA2R1 targeting miRNAs (*hsa-miR-23a, −23b, −23c, −141, −149, −154, −181b, −181d-1, −181d-2, −302d, −501*) (Table 2). Figure 4 shows how mean values of miRNAs expression varied in cancer cells and HMEC. According to these results miRNAs were differentially expressed in mammary cells with high (HMEC, CAL-51, and UACC-812 cells) and low (BT-474, MCF-7, and MDA-MB-453 cells) levels of PLA2R1 transcripts. Negative values of Pearson correlation coefficient, −0.628, −0.495, −0.468, −0.131, and −0.089 were observed for *hsa-miR-141, −181d-1, −181b, −501, and −149*, respectively (Table 2). In contrast, *hsa-miR-302d, −154 and −23b* levels positively correlated with receptor mRNA levels. To select miRNA candidates as possible additional regulators of PLA2R1 expression, we converted individual miRNA expression values into z-score that is considered as a useful tool to combine scores from data with different means, ranges, and standard deviations. Mean of z-values (mean z-score) allows the assessment of the multiple markers as a continuous variable [30]. In case of mean z-score of all miRNAs analyzed in this study, a weak negative Pearson correlation of this combined score with PLA2R1 expression was detected (*r* = −0.077, Table 1). Mean z-scores of five (*hsa-mir-141, −149, −181b, −181d-1, −501*) and three candidate miRNAs (*hsa-mir-141, −181b,* and *−181d-1)*, which all were negatively associated with PLA2R1 mRNA levels, showed a stronger correlation with the receptor expression (*r* = −0.581 and −0.721, respectively; Table 1). In case of mean z-score of *hsa-miR-23b, −154, and −302d*, a strong and significant positive correlation with PLA2R1 expression was found (*r* = 0.923, *p* = 0.009; Table 1).

**Table 2:** Expression of different miRNAs in normal HMEC and mammary cancer cell lines. Relative levels of miRNA were normalized to U6 snRNA level. Pearson product moment correlation coefficients (*r*) between PLA2R1 and miRNA expressions are shown. Databases: *a* - http://www.umm.uni-heidelberg.de/apps/zmf/mirwalk/predictedmirnagene.php; *b* - http://mirdb.org/cgi-bin/search.cgi; *c* - http://www.microrna.org/microrna

miRNAs	HMEC	CAL-51	UACC-812	BT-474	MCF-7	MB-453	r	Database
miR-23a	2.610 ± 0.065	1.920 ± 0.160	2.470 ± 0.011	3.020 ± 0.545	3.050 ± 0.080	0.925 ± 0.024	0.085	*c*
miR-23b	0.692 ± 0.333	1.620 ± 0.045	2.990 ± 0.245	1.080 ± 0.140	2.230 ± 0.055	0.268 ± 0.007	0.277	*c*
miR-23c	0.0066 ± 0.0625	0.0098 ± 0.0003	0.0148 ± 0.0625	0.0095 ± 0.0625	0.0199 ± 0.0005	0.0018 ± 0.0001	0.022	*c*
miR-141	1.810 ± 0.140	0.028 ± 0.009	3.780 ± 0.305	2.200 ± 0.120	4.910 ± 0.130	3.100 ± 0.080	−0.628	*a, b*
miR-149	0.0677 ± 0.012	0.0920 ± 0.0157	0.3040 ± 0.0170	0.0725 ± 0.0045	0.3690 ± 0.0100	0.0404 ± 0.0022	−0.089	*a, b, c*
miR-154	0.0163 ± 0.0004	0.0012 ± 0.0001	0.0024 ± 0.0002	0.0007 ± 0.0001	0.0008 ± 0.0001	0.0017 ± 0.0001	0.451	*a*
miR-181b	0.223 ± 0.023	0.820 ± 0.070	0.129 ± 0.004	0.359 ± 0.009	0.483 ± 0.039	1.780 ± 0.032	−0.468	*c*
miR-181d_1	0.0176 ± 0.0018	0.0007 ± 0.0001	0.0002 ± 0.0001	0.0004 ± 0.0001	0.0005 ± 0.0003	0.1070 ± 0.0086	−0.495	*c*
miR-181d_2	0.0002 ± 0.0001	0.0271 ± 0.0008	0.0039 ± 0.0013	0.0180 ± 0.0034	0.0162 ± 0.0005	0.0005 ± 0.0002	0.146	*c*
miR-302d	0.00022 ± 0.00002	0.00637 ± 0.00037	0.00016 ± 0.00009	0.00001 ± 0.00001	0.00007 ± 0.00003	0.00024 ± 0.00004	0.587	*a*
miR-501	0.0115 ± 0.0013	0.0312 ± 0.0018	0.0554 ± 0.0017	0.0064 ± 0.0006	0.0302 ± 0.0009	0.0570 ± 0.0131	−0.131	*a*

## Discussion

The findings of this study demonstrate that the PLA2R1 is differentially expressed amongst mammary normal and cancer cells, and confirm the importance of epigenetic mechanisms such DNA methylation and histone modification in the regulation of PLA2R1 transcript levels. Similar to previous data obtained in leukemia cells [12], the analyzed *PLA2R1* promoter region was nearly completely methylated in the mammary cancer cell line MDA-MB-453 where *PLA2R1* gene was silenced (Figs. 1 and 2). These findings are consistent with previous studies underscoring the role of DNA methylation in gene silencing mechanism critically involved in cell cycle regulation, carcinogen detoxification, cell adhesion and metastasis [31]. In addition to DNA methylation, the importance of histone modification in PLA2R1 regulation was indicated by the synergistic re-expression of PLA2R1 after simultaneous treatment of MDA-MB-453 cells with DNA methylation and histone deacetylase inhibitors (Fig. 2).

Recently, PLA2R1 was identified as potential tumour suppressor that controls replicative- and stress-induced senescence [9, 13, 14]. Normal cells employ cellular senescence to control or prevent carcinogenesis, while cancer cells suppress senescence to increase their survival capacity [9, 14, 32]. The importance of PLA2R1 for regulation of cell life span was confirmed in vivo, as *PLA2R1* knockout mice were more sensitive to RAS-induced skin tumours [10]. Conversely, constitutive expression of PLA2R1 in normal cells activated premature senescence [8, 13]. However, little is known about the pathophysiological significance of senescence during mammary carcinogenesis, although recent data indicated the involvement of senescence in the regulation of mammary cancer progression [32]. Whether the activation of senescence is linked to levels of PLA2R1 expression in the investigated subset of cell lines remains unclear. Therefore, it will be of interest for future studies to assess which cellular signalling pathways are activated by PLA2R1 and which physiological ligands trigger the PLA2R1 mediated signalling functions in mammary cancers.

The methylation analysis data we observed in MDA-MB-453, MCF-7 and BT-47 cells (Figs. 1 and 2) are consistent with previous unsupervised cluster analysis of methylation-sensitive gene expression [33]. The study revealed a subset of mammary cancer cells, including MDA-MB-453 cells, which were classified as hypermethylator cell lines and exhibited aberrant DNA hypermethylations of distinct genes. This cell line subset exhibited elevated DNMT activities. In contrast, mammary cancer cells classified as low-frequency methylator cell lines did not show increased methylation of specific genes or DNMT activity [33]. In agreement with these studies we detected an up-regulation of DNMT1 expression in MDA-MB-453, MCF-7, and BT-474 cells (Fig. 3) which are characterized by increased *PLA2R1* promoter methylations (Fig. 1). Whether this DNMT1 expression is causally connected to the observed *PLA2R1* promoter methylation requires further investigation.

A growing number of data confirmed that microRNAs play a crucial role in the regulation of gene expression *via* control of post-transcriptional mRNA function. As “global-regulators” miRNAs direct a diverse range of cellular responses both in normal and pathological conditions [20, 34]. Expression of miRNA is one of the universal epigenetic mechanisms implicated in the regulation of growth and survival pathways in cancer cells [35].

We identified a significant up-regulation of miR-141, miR-181b, and miR-181d-1 and a down-regulation of miR-23b, miR-154 and miR-302d in mammary cancer cells in comparison to HMEC. According to the applied databases (Table 2), these miRNAs exhibit extended complementarity to the 3′-UTR sequence of *PLA2R1* gene. Therefore, further studies are warranted to confirm regulatory effects of hsa-miR-23b, −154 and −302d (positive regulators) and hsa-miR-141, −181b, and −181d-1 (negative regulators) on PLA2R1 expression. The observed depletion of *hsa-miR-154* in all analyzed mammary cancer cell lines is a novel finding that should also be investigated in further detail.

Associations between miRNA expression and cancer progression were reported for different cancer types [20, 36], while selected miRNAs, that were analysed in this study, have been described as regulators of carcinogenesis. For instance, *hsa-miR-141* is overexpressed in cisplatin resistant ovarian, gastric and esophageal squamous cancer cells [37–39]. Furthermore, *hsa-miR-141* expression was associated with chemoresistance in breast cancer patients receiving neoadjuvant chemotherapy [40] and was elevated in MDA-MB-231 invasive breast carcinoma cell line [41]. Members of the *hsa-miR-181* family were involved in myeloid differentiation and acute myeloid leukemia [42]. It was noted that *hsa-miR-181a/b* overexpression coincided with aberrant activation of major signalling pathways involved in breast tumourigenesis, including IL6/STAT3 [43], TGF-β [44, 45], HIF-1 [46], WNT/β-catenin [47] and HMGA1 [48]. The *hsa-miR-181* family has been shown to be deregulated also in other solid tumours such pancreas, prostate, gastric and colon cancers and was able to target tumour suppressors, including TIMP3, CYLD, PTEN and p27 [43, 45, 49, 50]. Overexpression of *hsa-miR-181a/b* in breast cancers correlated with aggressive features and the likelihood to develop distant metastases [44, 51, 52]. Our findings are consistent with the observation that hsa-miR-181d-1 and −181b are strongly up-regulated in the metastatic MDA-MB-453 mammary cancer cell line (Fig. 4).

Among miRNAs, which were positively associated with PLA2R1 expression in this study, hsa-miR-23b and −154 exerted suppressing effects in different cancers *in vitro* and in vivo [53–55]. Consequently, it will be of special interest to elucidate the effects of distinct miRNA inhibitors on cellular PLA2R1 expression in future studies.

## Conclusions

The data of this study indicate that the PLA2R1 is differentially expressed in mammary normal and cancer cells and that the cellular receptor expression is regulated by epigenetic mechanisms such as DNA methylation and histone acetylation. An up-regulation of DNMT1 was found in cells with high *PLA2R1* promoter methylation. In addition, new candidate miRNAs such as hsa-miR-23b, −154 and −302d which are positive regulators and hsa-miR-141, −181b and −181d-1 which are negative regulators were identified. These miRNAs should be further tested as putative regulators of PLA2R1 expression in mammary cancer cells.

## Abbreviations

5-aza-dC: 5-aza-2′-deoxycytidine
DNMT: DNA-methyltransferase
FCS: Fetal calf serum
MS-HRM: Methylation-specific high resolution melting
PLA2R1: M-type phospholipase A_2_ receptor
RT-qPCR: Reverse transcription quantitative polymerase chain reaction
sPLA_2_: Secreted phospholipase A_2_
miRNA: Micro RNA
TSA: Trichostatin A
